# Opto-Avoidance-Elevated Plus Maze protocol to study positive or negative valence upon optogenetic stimulation in the mouse brain

**DOI:** 10.1016/j.xpro.2024.103474

**Published:** 2024-12-06

**Authors:** Gian Pietro Serra, François Georges, Åsa Wallén-Mackenzie

**Affiliations:** 1 Uppsala University, Department of Organism Biology, 752 36 Uppsala, Sweden; 2 Aligning Science Across Parkinson’s (ASAP) Collaborative Research Network, Chevy Chase, MD 20815, USA; 3 Université de Bordeaux, Institut des Maladies Neurodégénératives, UMR 5293, 33000 Bordeaux, France; 4 CNRS, Institut des Maladies Neurodégénératives, UMR 5293, 33000 Bordeaux, France; 5 Lund University, Department of Experimental Medical Science, Box 118, 221 00 Lund, Sweden

**Keywords:** Neuroscience, Cognitive Neuroscience, Behavior

## Abstract

The elevated plus maze (EPM) apparatus consists of two open arms that provide aversive spaces and two closed arms that provide protective and welcoming spaces. Here, we present a protocol to implement the classical EPM apparatus in a real-time optogenetic environment to address behavioral avoidance in mice. We describe steps for performing stereotaxic surgery, mouse manipulation, and experimental setup. Finally, we detail the procedures required for the Opto-Avoidance-EPM test in which the naturally preferred closed arms are selectively paired with optogenetic stimulation.

For complete details on the use and execution of this protocol, please refer to Serra et al.^1^

## Highlights

• Steps for stereotaxic adeno-associated virus injection and cannula implantation
• Procedures for mouse handling and habituation to the experimental environment
• Guidance on setting up the equipment, experimental room, and tracking software
• Detailed instructions on how to perform the Opto-Avoidance-EPM test

## Abstract (editor highlights)

Publisher’s note: Undertaking any experimental protocol requires adherence to local institutional guidelines for laboratory safety and ethics.

## Graphical abstract

**Figure:**
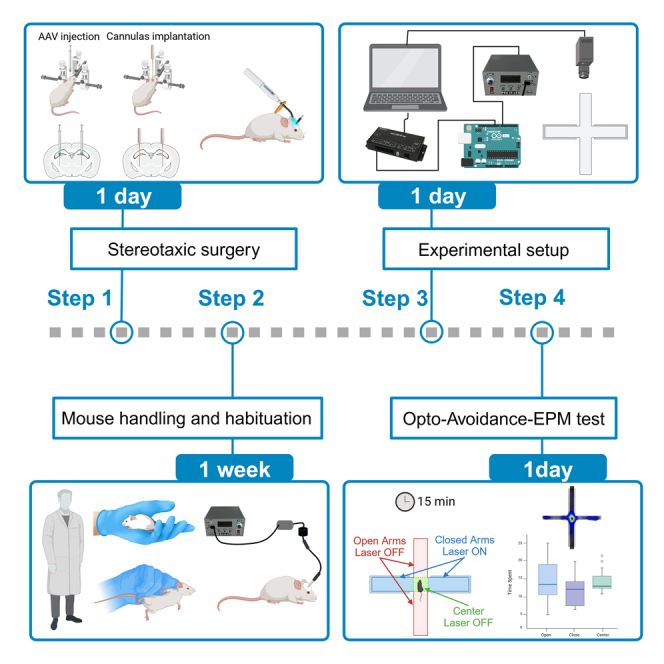

## Before you begin

The EPM consists of four arms elevated from the floor and connected via a central area. Two arms are open and exposed, providing a naturally aversive space in which mice avoid spending time. The other two arms are closed, providing a sheltered space that mice prefer over open and naturally aversive arms. The EPM test was developed to study anxiety.^2^^,^^3^ This is based on the fact that mice prefer the closed, sheltered arms and avoid the exposed open arms and center, thus showing avoidance towards the area which induces increased anxiety state. An increased time spent in the open arms compared to control mice is considered an indicator of anxiolytic effect.^2^^,^^4^

Here, we present a re-design of the classical EPM test. This new protocol is referred to as the Opto-Avoidance-EPM, and was developed and used by Serra et al.^1^ to evaluate any adverse effect of photostimulation of either the subthalamic nucleus (STN) or para-STN (pSTN) in mice in which Channelrhodopsin 2 (ChR2) was expressed in a Cre-dependent manner under the control of various promoters (promoters for Vesicular glutamate transporter 2 (Vglut2) and Paired-like homeodomain 2 (Pitx2) to drive selectivity towards the STN and Tachykinin precursor 1 (Tac1) to drive selectivity towards the pSTN).^1^ The Opto-Avoidance-EPM test exploits the conflicting behavior of mice between the tendency to explore a new area and the aversion to open and elevated spaces to estimate the extent of an aversive effect induced by optogenetics stimulation.^1^

Optogenetic stimulation can induce excitation or inhibition in a neuronal population selected based on the expression of light-sensitive opsins and the presence of a fiber optic cannula that provides photostimulation via a laser or LED light source.^5^^,^^6^

In the Opto-Avoidance-EPM test, entering one of the closed and protected arms is associated with photostimulation (Laser-ON). Entering the center or one of the open arms causes the photostimulation to be interrupted or remain switched off (Laser-OFF). Thus, by coupling the sheltered and naturally preferred EPM area with optogenetic stimulation, the Opto-Avoidance-EPM protocol allows for a direct comparison between a naturally aversive context (open arm) and the aversion caused by optogenetic activation (closed arm).

The Opto-Avoidance-EPM protocol can be adapted and used to study any brain area hypothesized to be associated with aversive or rewarding effect induced by optogenetic modulation.

The steps necessary to test the hypothesis that an optogenetic stimulation of choice results in an aversive effect that induces an avoidance response, are summarized below.

### Institutional permissions

All experimental procedures using mice followed Swedish (Animal Welfare Act SFS 1998:56) and European Union Legislation (Convention ETS 123 and Directive 2010/63/EU).

## Key resources table

**Table:** 

REAGENT or RESOURCE	SOURCE	IDENTIFIER
**Bacterial and virus strains**		

AAV2/EF1a-DIO-hChR2(H134R)-eYFP 3.8 × 10^12^ virus molecules/mL	UNC Vector Core, Chapel Hill, NC, USA and Dr. R. Jude Samulski, PhD	N/A ; Report Number: R46504
AAV2/EF1a-DIO-eYFP 4.6 × 10^12^ virus molecules/mL	UNC Vector Core, Chapel Hill, NC, USA and Dr. R. Jude Samulski, PhD	N/A; Report Number: R46503

**Chemicals, peptides, and recombinant proteins**		

Optibond FL 1 Prime	Kerr	Ref. 25881
Optibond FL 2 adhesive	Kerr	Ref. 25882
Isoflurane	Baxter Medical AB	N/A
Marcaine (bupivacaine 5 mg/mL)	Aspen Nordic	N/A
Rimadyl Bovis vet. (carprofen 50 mg/mL)	Zoetis Animal Health ApS	N/A
Jodopax vet. (iodine 5% and acetic acid 15%)	https://www.apoteket.se/	N/A

**Software and algorithms**		

EthoVision XT 16	Noldus Information Technology, the Netherlands https://www.noldus.com/	RRID:SCR_000441
GraphPad Prism version 7.00 for Windows	GraphPad Software, La Jolla, California, USA http://graphpad.com/	RRID:SCR_002798
Microsoft Excel 2019	Microsoft https://www.microsoft.com/en-gb/	RRID:SCR_016137

**Other**		

Optical power and energy meter console, Digital 4″ LCD PM100D	Thorlabs	N/A
Mono fiber-optic cannula	Doric Lenses	Cat#MFC_200/245–0.37_5.0mm_ZF1.25_FLT
Splitter branching fiber-optic patch cords	Doric Lenses	Cat#BFP(2)_200/240/900–0.22_1m_FCM-2xZF1.25
Mono fiber-optic patch cords	Doric Lenses	Cat#MFP_200/220/900–0.22_1m_FCM-ZF1.25
Elevated plus maze	Stoelting, http://www.stoeltingeurope.com	Cat#60140
MBL-III-473 nm-100 mW laser	CNI Lasers, Changchun, China	N/A
TTL Mini USB-IO box	Noldus Information Technology, the Netherlands	N/A
Laser safety glasses, light orange lenses, 48% visible light transmission LG3	Thorlabs	N/A
1 × 1 Fiber-optic rotary joints	Doric Lenses	Cat#FRJ_1 × 1_FC-FC
Mating sleeves	Doric Lenses	Cat#SLEEVE_ZR_1.25
Arduino UNO card	Arduino	RRID:SCR_017284
Stainless steel anchor screws 1 × 2 mm	AgnTho’s AB, Sweden	Cat#MCS1x2
Legato 130 syringe pump	KD Scientific	Cat#788130
NanoFil 10 μL syringe	World Precision Instruments	N/A
NanoFil injection needles 34G	World Precision Instruments	Cat#NF34BV
Induction chamber	AgnTho’s AB, Sweden	Cat#8329001
Gas routing switch	AgnTho’s AB, Sweden	Cat#8433005
Stereotaxic mask	AgnTho’s AB, Sweden	Cat#907
10 mL Gas tight syringe for the U-410 & U-1200 series	AgnTho’s AB, Sweden	Cat#7751001
Univentor 410 Anesthesia unit	AgnTho’s AB, Sweden	Cat#8323101
Ethicon Vicryl Rapid, 6–0, P-1 needle	AgnTho’s AB, Sweden	Cat#V32H

## Materials and equipment

The materials and equipment needed for the surgical phase of the protocol are present in the key resources table and their use is described in the "step-by-step method details" section. Below is a list of all the components needed to perform the Opto-Avoidance-EPM test.

• An Elevated Plus Maze apparatus for mice (Stoelting Co., USA).
• A video tracking software and a computer. We used EthoVision XT and the settings that follow refer to the use of this software (EthoVision XT 16, Noldus) but, in alternative, it is possible to use other video tracking software (Any-maze, BIO-EPM3C Bioseb, All Maze Orchid scientific, etc.).
• A hardware control module for the video tracking software. We used the Mini IO box (Noldus) that is connected, via USB, to the computer where the EthoVision XT software is installed. This device provides the interface between the EthoVision XT software and peripheral device (e.g., pulse generators or Arduino card) and generates a TTL signal when the conditions for the optogenetic stimulation are met (e.g., when the animal enters the closed arms).
• A laser or LED light source with the optimal wavelength for the opsin used in the experiment. We used a laser (MBL-III-473 nm-100 mW) that emits light at a wavelength of 473 nm, which is ideal for ChR2 activation.
• A pulse generator (for example Prizmatix Pulser, AMPI Master-9, Agilent Waveform Generator). Alternatively, an Arduino card can be used. We used Arduino UNO which was programmed to control the laser to emit light at a frequency of 20 Hz with 5 ms pulses (Data S1). The Arduino card (or the pulse generator) must activate the output device (laser or LED) when the ON signal is received, and until the ON signal is deactivated (for example when the animal comes out of the closed arms).
• Fiber optic that must be connected to the laser or LED light source. We used a patch cord that evenly splits the light into 2 branches of optical fibers in order to achieve bilateral stimulation (Splitter Branching Fiber-optic Patch Cords FRJ_1 × 1_FC-FC, Doric Lenses).
• A rotary joint to use as a part of the fiber-optic tether to allow freely animals' movements. We used 1 × 1 fiber-optic rotary joint (Doric Lenses).
• Mating sleeves (we use sleeves suitable for 1.25 mm ferrules, Doric Lenses) to connect the patch cord to the cannulas implanted on the animal’s head.
• A video camera to be positioned vertically for overhead shooting. This camera must be connected to the computer where the video tracking software is installed.
• A second video camera, preferably equipped with a tripod, which can be oriented horizontally for lateral shooting.
• Optic fiber holder. We use metal support with clips to hold the rotary joint in place.

## Step-by-step method details

### Stereotaxic adeno-associated virus injection and cannula implantation

**Timing: 1 day**

This step serves to prepare the mice for *in vivo* optogenetic stimulation. Opsins will be expressed locally in the area where the injection is performed. In the control group, only the fluorescent protein YFP is expressed but not the opsin so that the neurons are not excited or inhibited when exposed to light. In this regard, if it is necessary to further restrict the expression of opsin and YFP protein (or YFP protein alone in controls) to a specific neuronal subpopulation, it is important to use a Cre line that is representative of the neural population of interest.

1. Stereotactic surgery.
  a. Fill a 10 mL syringe (10 mL gas-tight syringe, AgnTho’s AB) with isoflurane and insert it into the anesthesia unit (Univentor 410 anesthesia unit, AgnTho’s AB).
    ***Note:*** Any type of anesthesia unit or system can be used for this purpose.
  b. Turn on the anesthesia unit and set it to 4% (isoflurane in air).
  c. Place the animal inside the induction chamber (Induction Chamber, AgnTho’s AB) connected to the anesthesia unit.
  d. Once the mouse is anesthetized, reduce the percentage of isoflurane to approximately 2% and switch the anesthetic flow to the mask installed on the stereotaxic frame (Stereotaxic Mask, AgnTho’s AB) via a gas routing switch (Gas Routing Switch, AgnTho’s AB).
  e. Place the animal in the stereotaxic frame.
  f. Cover the animal’s eyeballs with an eye ointment to protect the cornea.
    ***Note:*** In our laboratory we use Bepanthen eye ointment (Bayer Leverkusen, Germany). Alternatively, any eye drops that are thick enough to protect the animals' corneas can be used (e.g., Hypotears, Lubrithal Eye Gel).
  g. Give the animal an injection of Carprofen 5 mg/kg s.c.
  h. Gradually reduce the isoflurane concentration from 2% to approximately 1.5%.
    **CRITICAL:** Monitor the animal's breathing and body temperature closely and ensure that it is properly anesthetized before beginning surgery.
  i. Expose the skull and perform the craniotomy.
    i. Carefully remove hair from the surface of the head.
    ii. Decontaminate the skin with antiseptic iodine solution (Jodopax vet).
    iii. Inject the local anesthetic bupivacaine hydrochloride (Marcaine) s.c. at the site where the surgical incision of the skin is to take place (wait 5 min until the local anesthetic takes effect).
    iv. Incise the skin with the scalpel until lambda and bregma are clearly visible on the surface of the skull.
    v. Look for the sagittal vein under the microscope and define your midline.
    vi. Verify that the skull is perfectly oriented on the horizontal plane.
    vii. Move to the bregma and zero all the coordinates, then move above the coordinates where you want to inject.
    viii. Make marks and drill the hole/s for virus injections and cannulas implantation.
    ix. Drill two additional holes of about one millimeter in diameter (one on each side posterior or anterior to the holes where the cannulas will be implanted) and insert a stainless-steel anchor screw into each hole.
      ***Note:*** To insert the screw use tweezers and a small screwdriver. We use stainless steel screws with a diameter of 1 mm and a length of 2 mm (AgnTho’s AB, Sweden).
      **CRITICAL:** Be careful not to go too deep when screwing in the screw (about half a millimeter).
      ***Optional:*** The screws can be marked before surgery to indicate half a millimeter from the tip.
      ***Note:*** This step is recommended but not mandatory. In fact, other dental cements (e.g., Metabond or Super-Bond) that do not require screws can be used as an alternative.
  j. Virus injection.
    i. Place the syringe (NanoFil 10 μL Syringe with NanoFil injection needles 34G, World Precision Instrument) in the syringe holder, which was previously screwed on the stereotaxic frame and connected to the automatic pump system (Legato 130 Syringe Pump).
    ii. Withdraw 1000 nL saline then 750 nL air and the desired amount of virus inside the syringe.
      ***Note:*** We recommend injecting a volume between 100 and 500 nL of virus depending on the size of the target area.
    iii. Before injecting, puncture the dura mater using a 32G needle with a bent tip.
    iv. Move the syringe to the designated coordinates (proceed very slowly when moving the syringe along the dorsal-ventral axis).
    v. Infuse the virus at a rate of 100 nL/min (for volumes between 100 and 500 nL).
    vi. After injection wait 10 min before lifting the needle.
    vii. Repeat on the contralateral side.
  k. Optic cannula implantation.
    i. Place the holder with the cannula on the stereotaxic frame and re-take the coordinates from bregma.
    ii. Before moving on the dorsal-ventral axis apply a mix of the primer (Optibond FL 1 Prime) and adhesive (Optibond FL 2 Adhesive) thoroughly on the skull, around the holes, the screws and on top of the screws.
      **CRITICAL:** Do not apply the mix on the holes.
    iii. Apply UV light for 20 s twice to harden the mix.
    iv. Implant the first cannula at the designed coordinates.
      ***Note:*** Cannulas can be implanted at the same coordinates where the virus injection was performed or at different coordinates (for example, if stimulation of the terminals in a target area different from the injection area is required).
    v. Before releasing the cannula, apply as much dental cement as possible around the cannula and screw.
      ***Note:*** Be careful to not interfere with the contralateral hole.
      **CRITICAL:** It is essential to leave a portion of the cannula uncovered by the cement so that the sleeve can be inserted correctly. The sleeve must be able to be inserted onto the cannula for at least half of its length. This is important to ensure that there is a perfect connection between the cannula and the optical fiber.
    vi. Apply the UV light for 20 s.
      **CRITICAL:** Be sure the optic cannula is well fixed before releasing it from the holder.
    vii. Repeat on the contralateral side.
    viii. Finish building the complex for the cannulas by covering them as much as possible having a smooth, round looking complex.
      **CRITICAL:** Make sure to leave no sharp edges which could hurt the mouse.
    ix. Apply the UV light for approximately 60 s to sufficiently harden the cement complex.
  l. End of surgery and recovery.
    i. Suture the posterior aspect of the incision and apply antiseptic iodine solution (Jodopax vet).
      ***Note:*** Additional sutures may be required on the anterior aspect of the incision. Sutures used should be absorbable (e.g., Vicryl, Dexon).
    ii. Give the mouse a subcutaneous saline injection (1 mL) and remove it from the stereotaxic frame.
    iii. Allow the mouse to recover from the surgery in a cage alone.
      ***Note:*** In the immediate postoperative phase, and until the animal has regained full consciousness, it is kept in a cage heated by a heating pad or alternatively under a heating lamp. We place half of the cage over (or under in case of a heating lamp is used) the heat source to help maintain normal average body temperature. During this phase, the animal is kept under close observation until it regains the righting reflex. Animals are reunited with their cage mates only after they can walk and move normally.
    iv. Inject the mice with Carprofen 5 mg/kg s.c every 24 h for a total of 2 consecutive days after the surgery.
    v. Monitor animal’s recovery during the following weeks.

### Mouse handling and habituation

**Timing: 3 weeks after surgery (1 week)**

Handling of the mice is an important step to habituate them to the experimenter, to the instruments to which they will be connected (optic cannula, laser, etc.), and to the room in which the experiment will be conducted (background noises, smells, lighting, etc.).

2. Experimenter manipulation to reduce stress.
  a. Day 1: Wear gloves and place one hand inside the cage without actively touching the mice but allowing the mice to interact with the hand.
  b. Day 2: Take the mice out of their cage one at a time and hold them on your arms for about 2 min. The animals are free to explore the experimenter’s arms and hands.
  c. Day 3: Take the mice out of their cage and hold them on your arms and hands for about 2 min. At this stage, the experimenter encourages the mouse to move from the forearm to the palm of his hand.
  d. Day 4: Take the mice out of their cage and place them directly on the palm of your hand. Hold the mouse on your hands for about 2 min.
    Before putting it back in the cage, restrain the animal as if you were to connect it to the optic fiber. Do not connect the optic fiber yet.
    **CRITICAL:** The same person who will perform the experiment must carry out the handling to reduce anxiety and novelty. Perform the handling in the same room where the experiment will be carried out in order to accustom the animal to both the experimenter and the room. The experimenter wears gloves during all phases. Gloves are changed between animals.
    **CRITICAL:** Allow the mice to acclimate to the room for at least 30 min before the experimenter begins handling them.
    ***Note:*** The lighting conditions in the room must remain consistent between habituation and testing. In our study we maintained a light intensity in the experimental room of approximately 300 Lux.
  e. Day 5: Restrain the animal with one hand and connect the optic fibers with the other hand. Place the animal in a neutral cage with the optic fiber connected for 5 min.
    **CRITICAL:** From this moment, throughout the habituation phase, a laser device will be kept on (without sending light) so that the animal can get used to the noise and this does not represent a novel factor during the tests.
    ***Note:*** The neutral cage is a clean cage with some bedding and enrichment from the home cage. Use the same cage for the next two habituation days and during the test day. It is recommended to mark the cage with the animal ID or IDs if used also for littermates.
  f. Day 6: Repeat the procedure and place the animal in its neutral cage with the optic fiber connected for 10 min.
  g. Day 7: Repeat the procedure and place the animal in its neutral cage with the optic fiber connected for 15 min.

### Experimental setup

**Timing: 1 day**

Here we describe how to arrange the necessary equipment in the experimental room and how to set up the software for tracking the animal’s movements and controlling the optogenetic stimulation.

3. Set up the room for the experiment:
  a. Place the apparatus exactly below the camera positioned on the ceiling to have a view from above of all four arms of the apparatus (Figure 1A).
    ***Note:*** The elevated plus maze apparatus used for mice has the shape of a plus sign; composed of four arms 35 cm long: two arms are open so that the animal can lean over the edges of these (“head dips”) and two closed with 15 cm high walls. The four arms intersect in the center to create a central platform (Figure 1A). The entire platform is raised 50 cm from the ground by four legs (Figure 1B). The two open, exposed arms provide a naturally aversive space that mice avoid. The other two arms are enclosed by walls on three sides, providing a sheltered space compared to the open arms representing more comfortable areas. The central area is less exposed than the open arms, but not sheltered (Figure 1A).

**Figure 1:**
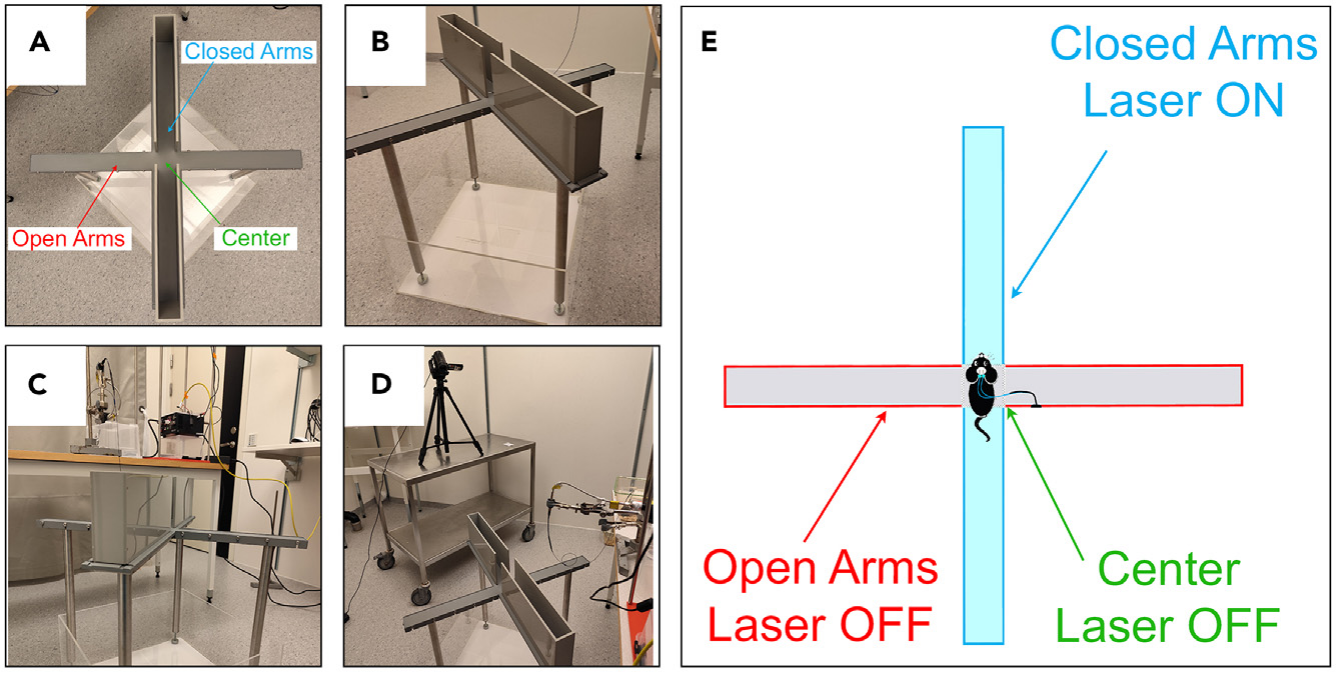
Experimental room setup (A) Top view of the four arms of the EPM apparatus. (B) Side view of the EPM apparatus. (C) Position of the laser device, Arduino card, fiber optic support and neutral cage with respect to the EPM apparatus. (D) Position of the second camera for the horizontal framing. (E) The closed arms are coupled with the activation of the laser device while the open arms and the center of the apparatus are coupled with the deactivation of the laser device.
  b. Mount walls on closed arms.
  c. Near the apparatus, place a table which will be necessary to place the laser (or LED) device, the Arduino card, the holder for the optical fiber and a cage where to place the animal after connecting it to the optical fiber (Figure 1C).
  d. Position a second camera horizontally to frame the center of the arena and film the animal’s entry into the closed arms from a different perspective (Figure 1D).
    ***Note:*** This is useful during analysis to check if there is any anomalous behavior or problems during the execution of the test. The animal may have difficulty moving due to a twisted fiber; the animal may increase vertical movements (rearing) while exploring the entrance of the closed arms; the animal may perform repeated movements in and out of the closed arms. With the horizontal framing you can more easily identify and better analyze this type of behaviors.
4. Set the video tracking software for tracking the animal’s movements so that entry into the arms coupled with optogenetic stimulation produces real-time activation of the light-emitting device (Figure 1E). Below are the steps to follow to set up EthoVision XT 16:
  a. Under “Experiment settings” select three points (nose, center and tail base) on “tracked features”.
  b. Set the port of the TTL Mini USB-IO box that is connected with the Arduino card under “Trial Control Hardware”.
  c. Under “Arena Settings” define the different areas and size (Figure 2).
    ***Note:*** To do this it is necessary to divide the apparatus into different areas: Closed arms; Open arms and central area. It is also advisable to define two "head dip zones" around the open arms to be able to identify any increases in exploration behaviors induced by optogenetic manipulation.

**Figure 2:**
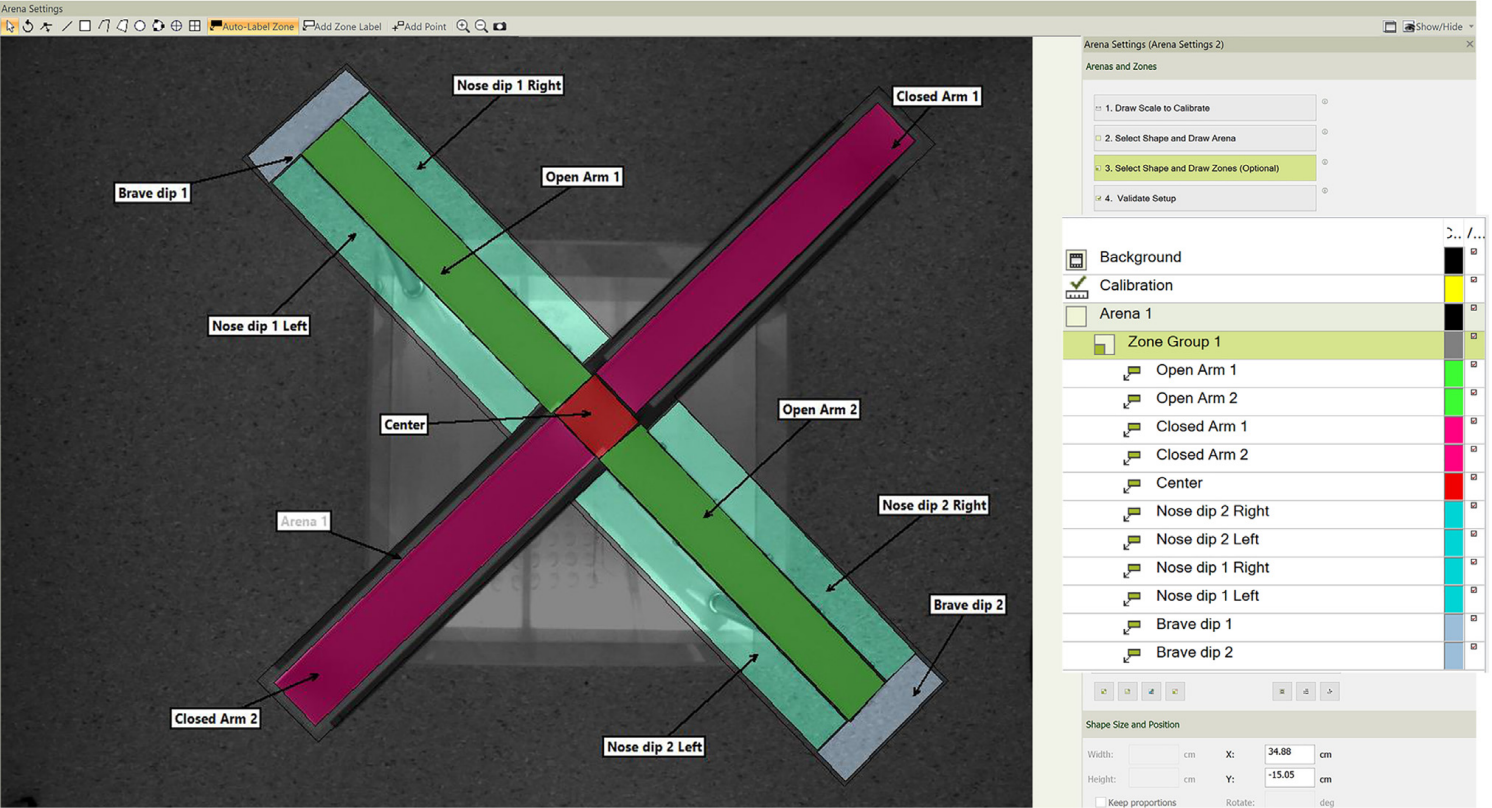
Arena settings on EthoVision
  d. Under “Trial control settings” create a Condition so that the experiment starts automatically 2 s after the mouse has been placed in the center zone. To do so select “Condition is met when center point is in Arena > 2 s” (Figure 3A).

**Figure 3:**
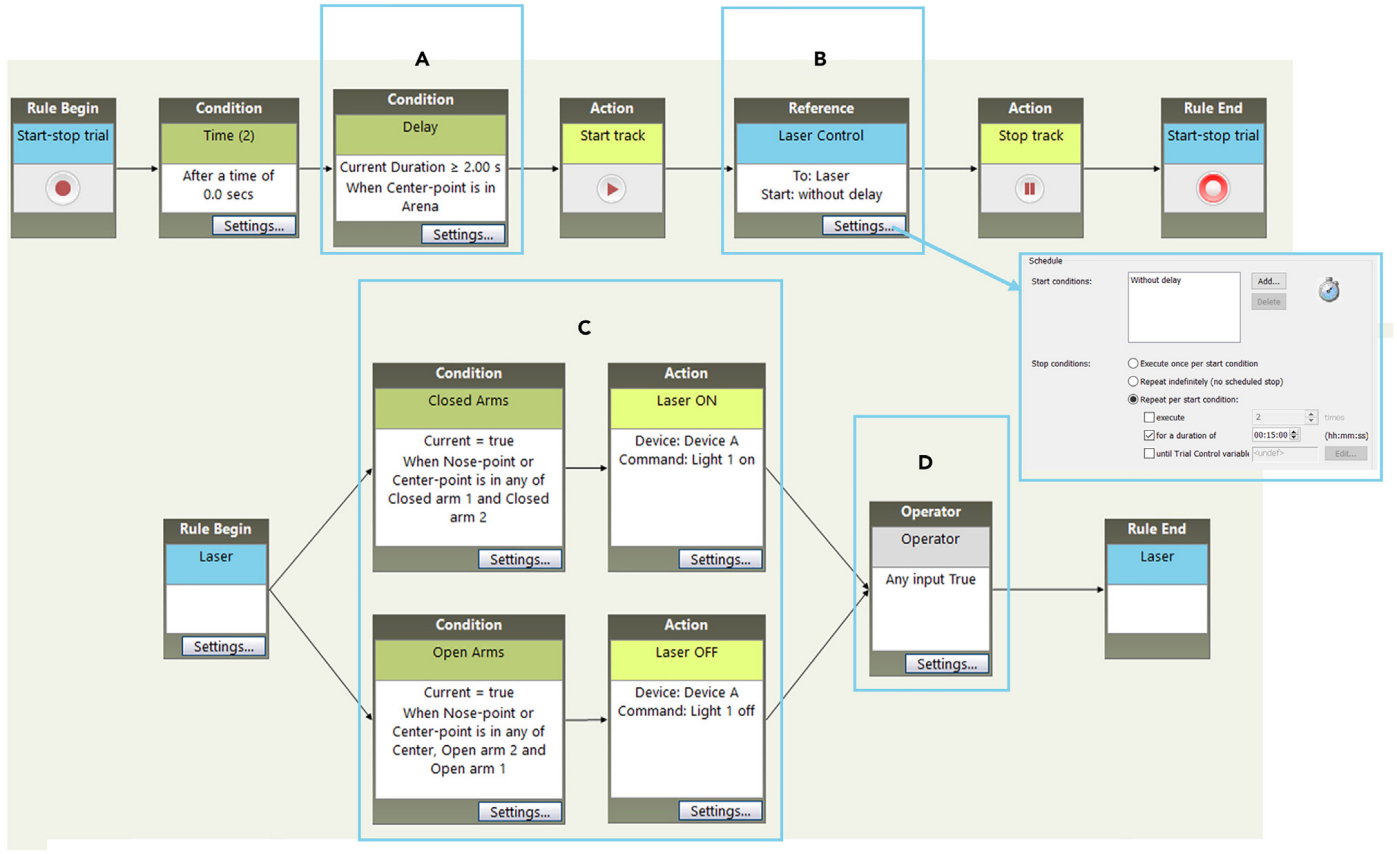
Trial control settings on EthoVision (A) Condition for the experiment to start automatically 2 s after the mouse is placed in the central zone of the apparatus. (B) Reference settings needed to define the duration of the test and to link the sub-rule to control the laser device. (C) Sub-rule settings so that the laser device turns on only when the animal enters the closed arms. (D) Setting for the Operator to be activated when any of the inputs is 'true'.
  e. Create a Reference (needed to control the Sub-Rule that will be created later). Open the “settings” menu of the Reference and rename it “Laser Control”. In the Schedule section select “without Delay” as start condition and “repeat for start condition” as stop condition; check the box “for a duration of” and select a duration of 15 min (Figure 3B).
    ***Note:*** Although a 5–10-min recording is common when performing the classic test, in this protocol the behavior is recorded for 15 min to allow the animal to create an association between the action of entering the closed arms and the effect induced by optogenetic stimulation.
  f. Create a Sub-Rule so that the laser will turn on when the animal enters the closed arms (Figure 3C).
  g. Create a Condition called “Closed Arms” selecting “condition is met when center point or nose point is in any of closed arm 1 and closed arm 2” (Figure 3C upper left).
  h. Create an Action called “Laser ON” to send the TTL to the Arduino card that in turn controls and turn on the laser and link this to the previous created condition (Figure 3C upper right).
  i. Create a second Condition called “Open Arms” selecting “condition is met when center point or nose point is in any of center, open arm 1 and open arm 2” (Figure 3C bottom left).
    ***Note:*** When creating a Condition in the Sub-Rule it is important to select both the center point and nose point to control the activation and deactivation of the laser. This is important because sometimes when mice are in the center area they move backwards and this may result in the mice actually being in a protected closed arm without activating the laser if only the nose is left out. Selecting the central point to activate the laser induces the animal to associate entering the closed arms with the stimulation, regardless of whether the animal enters frontally or moving backwards. It is important not to select the base-tail point because this part of the body could accidentally end up inside the closed arms during simple orientation maneuvers within the central area. In this case, inappropriate activation of the laser would only confound the animal.
    ***Note:*** In order to avoid prolonged periods of stimulation, it is advisable to program a maximum laser on time. For example, a protocol of 10 s on and 5 s off would not interfere with the conditioning effect of the stimulation and at the same time would protect the integrity of the neuronal cells. The activation duration of the light source (Laser or LED) can be programmed by changing the duration and intervals of the TTL signal (on the video tracking software), or by programming the pulse generator (if available).
  j. Create a second Action called “Laser OFF” to turn off the laser when the animal leaves the closed arms and link this Action to the previous created Condition (Figure 3C bottom right).
  k. Create an Operator and select “Operator triggers when any (or at least one) of the input is ‘true’” (Figure 3D).
  l. Place a mouse to be used for calibration inside the apparatus. Under “Detection Settings” you can select whether the animal is darker or lighter than the background. Choose “Automated setup” to automatically set the parameters that allow effective tracking of the animal. If necessary, these setting can be modified later under “Advanced settings”.
    ***Note:*** The mouse used for detection calibration must not be an experimental animal. It is advisable to use a littermate animal.

### Opto-Avoidance-EPM test

**Timing: 1 day**

In this section we explain how to set up and connect all the equipment needed to run the test (laser, fiber optics, etc.) and how to run the test.

5. Preparing the equipment.
  a. Connect the laser to the fiber optic through a rotary joint (Figures 4A and 4B).
    ***Note:*** We use 1 × 1 fiber-optic rotary joints (Doric lenses) to ensure free rotation of the fiber following the movements of the animal.
    **CRITICAL:** The use of the rotary joint is important to prevent the fiber from twisting when the animal turns on itself.
    ***Note:*** As a light source we use the MBL-III-473 nm-100 mW laser (CNI Lasers, Changchun, China), which includes a patch cord with FC connector metal ferrule output, that ensures direct connection to the rotary joint. Alternatively, an additional connection cable may be needed to connect the device used as a light source to the rotary joint (the type of cable connectors may vary depending on the device used).

**Figure 4:**
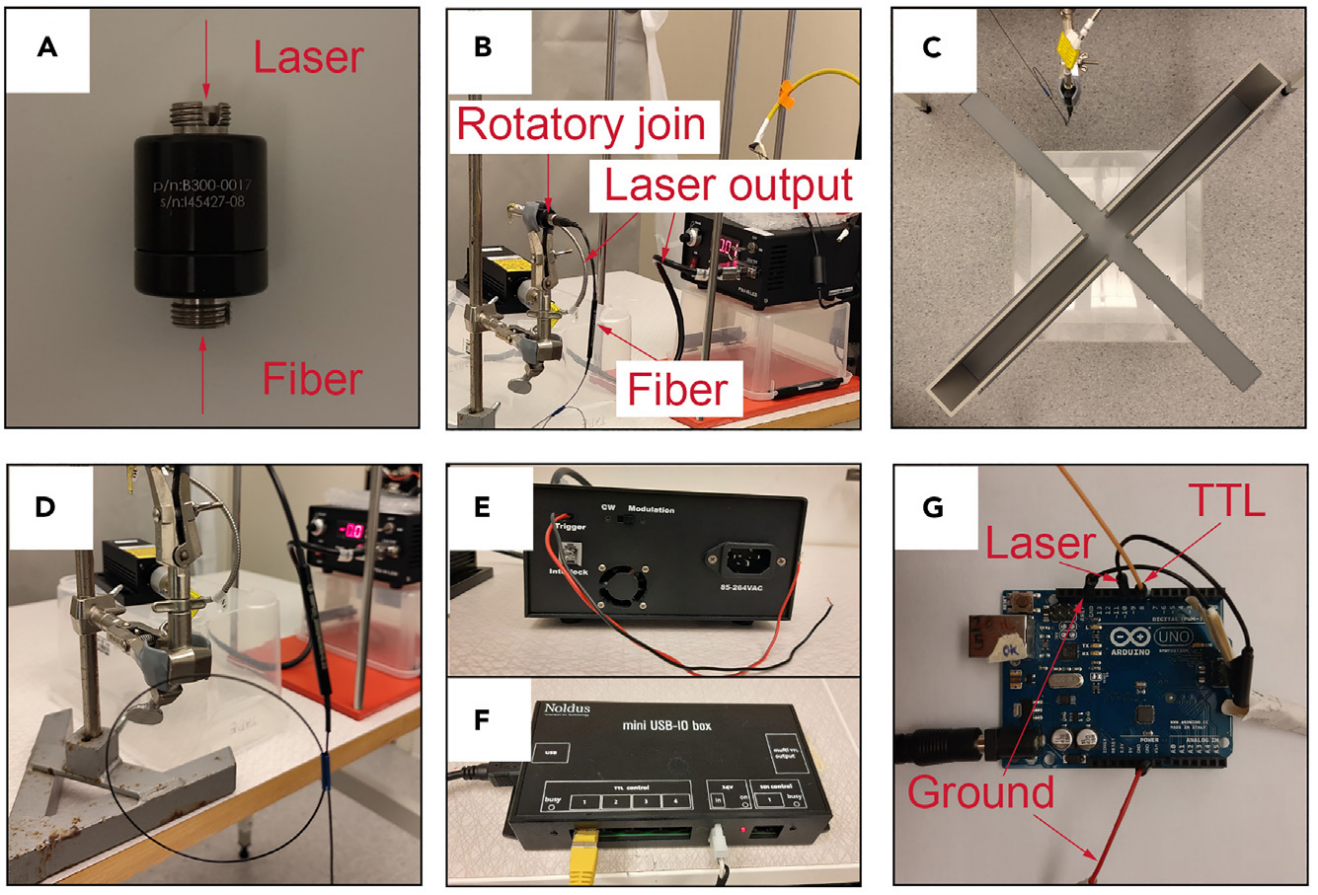
Equipment connections for optogenetic stimulation (A) Rotary joint. (B) Connections between the laser device, the rotary joint and the optical fiber. (C) Positioning of the rotary joint and the rotary joint support in relation to the EPM apparatus. (D) Loop that allows the optic fiber to be extended in case the animal falls from the apparatus. (E) Rear view of the laser device; the wires that are connected to the Arduino card are shown. (F) TTL Mini USB-IO box; the yellow cable is connected to port 1 to send the TTL signal to the Arduino card. (G) Arduino UNO card; the wiring between the laser device, the TTL Mini USB-IO box with the Arduino card is shown.
  b. Fix the rotary joint to an arm with a clip that holds it still and position it near the apparatus in such a way as not to obstruct the view of the cameras (Figure 4C).
  c. Connect a fiber optic patch cord to the other end of the rotary joint (Figures 4A and 4B).
    ***Note:*** We use 1 m long splitter branching fiber-optic patch cords (Doric Lenses) equipped with FC connector with metal ferrule on the end that is connected to the rotary joint and two 1.25 mm zirconia ferrule terminals on the end that must be connected to the optical cannulas implanted on the animal. Alternatively, two mono fiber-optic cannulas (Doric Lenses) connected to a 1 × 2 fiber-optic rotary joint (Doric Lenses) can be used.
    **CRITICAL:** The optical fiber must be at least 1 m long. This is important because it could happen that the mouse falls after leaning out of one of the open arms. If this happens and the optical fiber is too short, this will result in the mouse hanging by its head. On the other hand, to prevent a fiber that is too long from hindering movement or getting stuck somewhere in the apparatus, it is important to form a loop that makes the fiber shorter so as not to disturb the animal's movements but at the same time allow the fiber to stretch in the event of a fall from the apparatus (Figure 4D).
    ***Note:*** If unilateral stimulation is performed, it is necessary to use single-terminal patch cords (for example Mono Fiber-optic Patch Cords, Doric Lenses).
  d. Connect the mating sleeves to the two ends of the optical fiber so that only half is inserted into the fiber ferrule.
    ***Optional:*** Place the apparatus inside a box made of transparent material, so that it does not interfere with the detection of the video tracking software, but prevents the mice from moving away from the surrounding perimeter in the event of a fall (Figure 4C). This is important to ensure that the mice do not exert force on the fiber and the surgically applied complex as they attempt to move away.
    ***Optional:*** The bottom of the box can also be covered with soft material that can cushion the fall.
    ***Note:*** These precautions are important to take, but it is important to note that, in our experience, only in very rare cases we observed mice falling from the apparatus.
  e. Connect the laser (Figure 4E) and the TTL Mini USB-IO box (Figure 4F) to the Arduino card which has previously been programmed to receive the TTL input on channel 8 and the laser output on channel 10 (Figure 4G and Data S1).
  f. Turn on the light source and adjust the intensity to 5 mW (if using opsins other than ChR2, different light intensities may be needed).
  g. Measure the intensity of the light coming out of each end of the optical fiber after connecting an optical cannula identical to those implanted in animals.
    ***Note:*** Use a power meter to measure the light intensity (e.g. Optical Power and Energy Meter Console, Thorlabs).
    **CRITICAL:** To ensure the same light intensity, repeat the operation between sessions.
    **CRITICAL:** Wear laser safety glasses during this operation to protect your eyes from the light source (e.g., Laser Safety Glasses Light Orange Lenses LG3, Thorlabs).
6. Test execution:
  a. Transfer the animals to the room where the experiment is performed.
    **CRITICAL:** The test must be performed during the same time frame (e.g. 10:00 am to 3:00 pm) as the habituation phase and must remain consistent for all subjects in the study.
    **CRITICAL:** Before the test, mice should be acclimatized in the experimental room for at least 30 min.
    ***Note:*** Place the home cages in the same area of the room as during the habituation phase and place a curtain, if the room has one, so that the area where the test takes place is somewhat isolated from the surrounding environment.
  b. Take the mouse from its cage and connect it bilaterally to the patch cord. Restrain the mouse with one hand and with the other hand connect the ends of the patch cord to the optic cannulas implanted on the animal (Figure 5A).
    ***Note:*** Use the sleeves that were previously connected on the ends of the patch cord making sure that the two ferrules are in perfect contact with each other.

**Figure 5:**
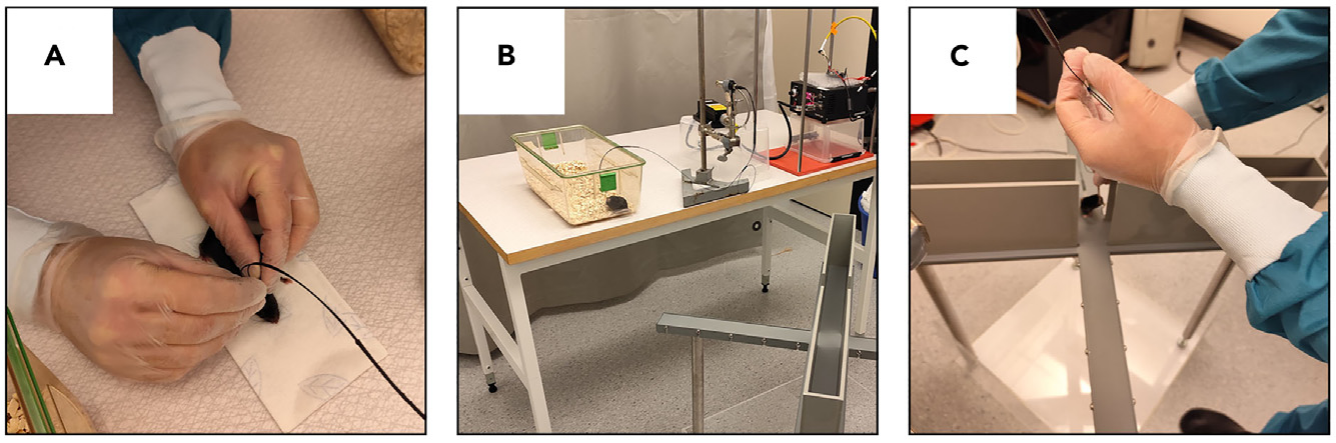
Connection of the optical fiber on the mouse and placement in the EPM apparatus (A) Restraining of the mouse to ensure the connection between the optical fiber and the cannulas. (B) Positioning of the mouse in the neutral cage to recover after the optical fiber connection. (C) Positioning of the mouse in the center zone of the arena facing one of the open arms.
  c. After connecting the mouse to the patch cord, place it in its neutral cage for 5 min to ensure consistent recovery time between animals before starting the test (Figure 5B).
    **CRITICAL:** The neutral cage has to be the same used during the habituation days.
  d. Start recording with the horizontal camera.
    ***Optional:*** After starting recording, display a card identifying the session and the animal ID, as reported in the video tracking software, for record keeping.
  e. Start acquisition on the video tracking software (EthoVision XT 16).
  f. Place the animal in the center zone of the arena facing one of the open arms (Figure 5C).
    ***Note:*** We position all animals facing the same arm, but it is also possible to alternate arms between sessions.
  g. Leave the room and follow the progress of the experiment through a screen in another room.
    ***Note:*** In our laboratory, the computer is located in a room adjacent to the one where the experiment is performed. In the experimental room only an auxiliary screen, a USB socket, keyboard and mouse are present (connected via wiring). Alternatively, if the computer is located in the experimental room, a remote access software (TeamViewer, AnyDesk, etc.) can be used to follow the progress of the experiment from another room.
    **CRITICAL:** Make sure the camera positioned on the ceiling is set with optimal focus and light exposure. This is important for proper tracking by the video tracking software.
    **CRITICAL:** The apparatus should be cleaned with a 70% ethanol solution and dried thoroughly between mice.

## Expected outcomes

In this test, the presence of the animal in the closed arms of the apparatus is coupled with photostimulation (Laser ON), while the presence in the open arms and in the central area determines turning off of the photostimulation (Laser OFF) (Figure 1E).

By applying the photostimulation in the closed arms, it is possible to compare the naturally aversive stimulus represented by open arms with the hypothesized aversive effect caused by the photostimulation (closed arms).

By applying this protocol, a sufficiently powerful aversive effect is expected to be able to induce active avoidance of the closed arms (normally preferred in mice) coupled to photostimulation, resulting in an increased time spent in the center and open arms. An increase in time spent either in the center or in the open arms, compared to controls, will be considered an index of avoidance towards the effect induced by optogenetic stimulation.

Additional note: This protocol can also be adapted to evaluate a rewarding rather than an aversive effect. In this case, it is necessary to change the area of the apparatus coupled with the optogenetic stimulation. For example, if it is hypothesized that photostimulation can have a positive valence; this will be applied upon entry into the open arms (normally avoided). If the optogenetic-induced effect is sufficiently rewarding, it is expected to be able to reverse the natural avoidance toward the open arms and make the animal spend more time exploring them.

## Quantification and statistical analysis

Export the recorded data from the video tracking software (e.g., time spent in arms, number of entries into different areas of the apparatus, etc.) to an Excel file. For statistical analysis and graph plotting, we used GraphPad Prism software version 10.0.0 for Windows (GraphPad Software, Boston, Massachusetts USA). Alternatively, other similar software can be used for statistical analysis and graph plotting (e.g., Posit, JMP, OriginPro, Stata, etc.). To compare the time spent by control and opsin-expressing mice in the three zones (open arms, closed arms and center), it is recommended to perform repeated measures two-way ANOVA followed by Šídák multiple comparisons analysis. EthoVision XT 16 provides the results both in absolute terms (such as total duration or number of entries) and in percentage (for example, the percentage of time spent in each area). Both types of data can be used as provided by the program for statistical analysis and graphical representation. We prefer to represent the time spent in each area of the apparatus as a percentage of the total time.

Exclude mice from analysis if post-hoc histology reveals insufficient eYFP expression or if cannulas position appears off-target. Exclude mice if histological analysis is not possible due to premature death of the mouse or if the mouse had to be sacrificed for health reasons during the study.

## Limitations

A limitation of this protocol is that the aversive (or rewarding) effect evoked by optogenetic modulation must be sufficiently powerful to counteract the innate preference that mice have for closed arms. It could happen that the aversive effect is not sufficiently powerful to evoke a significant response, but is still experienced by the animal as an aversive event. For example, stimulation of a small neuronal subpopulation might cause avoidance in a real-time conditioned place preference test,^7^ but not in the Opto-Avoidance test presented here.^1^ The authors therefore recommend including a battery of tests to be carried out to ascertain the nature of any observed aversive effect.

## Troubleshooting

### Problem 1

In some cases, it may happen that the tracking signals conflict with background noise, for example when the animal is in more shaded areas (closed arm ends). Related to Step 3.

### Potential solution

Since the behavioral tracking system is based on light contrast (for example the difference between the animal’s color and the background color) it is important to set the correct light exposure of the camera. We have found that increasing the light exposure level of the camera connected to the video tracking software reduces the occurrence of this problem.

### Problem 2

The optical fiber twists even if connected to the rotary joint, preventing correct movement of the animal inside the apparatus. Related to Step 4.

### Potential solution

**Figure 6:**
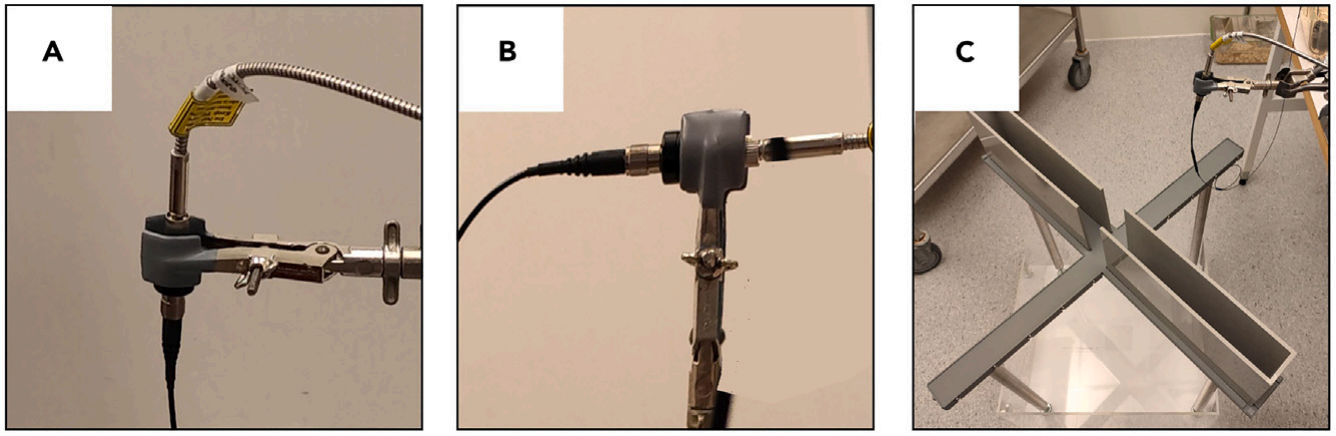
Troubleshooting fiber twisting (A) Rotary joint and optical fiber with vertical orientation. (B) Rotary joint and optical fiber with horizontal orientation. (C) Positioning of the rotary joint and optical fiber complex relative to the EPM apparatus.
• A second precaution is to bring the rotary joint as close as possible to the center of the apparatus without positioning it exactly above it to avoid disturbing the detection of the animal by the camera oriented vertically for the shot from above (Figure 6C).

• We have observed that orienting the rotary joint so that the optical fiber has a vertical orientation (Figure 6A) drastically reduces the possibility of the fiber becoming twisted. The vertical orientation (Figure 6A) is therefore preferable to the horizontal orientation (Figure 6B).

### Problem 3

During the test the patch cord gets completely or partially disconnected. This problem can occur if the connection between the optical fiber and the cannula has not been made correctly. Related to Step 4.

### Potential solution

• To obtain a better grip of the sleeve on the ends of the two ferrules (the one at the end of the optical fiber and the one on the cannula) it is important to ensure that the sleeve has entered half of its length onto the two ferrules and that these are in perfect contact with each other.
• Make sure there are no obstacles where the fiber can get stuck.

## Resource availability

### Lead contact

Further information and requests for resources and reagents should be directed to the lead contact, Åsa Wallén-Mackenzie (asa.mackenzie@med.lu.se).

### Technical contact

Technical questions on executing this protocol should be directed to and will be answered by the technical contact, Gian Pietro Serra (gianpietro.serra@ebc.uu.se).

### Materials availability

This study did not generate new unique reagents.

### Data and code availability

The Arduino UNO programming code is provided as a Supplemental File.

## Acknowledgments

This study was funded by Vetenskapsrådet, Hjärnfonden, Parkinsonfonden, Research foundations of Bertil Hållsten, Zoologiska, and Åhlén (Å.W.-M.); and Research foundations of Zoologiska and OE & Edla Johansson (G.P.S.). The study was also funded by the joint efforts of The Michael J. Fox Foundation for Parkinson’s Research (MJFF) and the Aligning Science Across Parkinson’s (ASAP) initiative. MJFF administers the grant (ASAP-020600) on behalf of ASAP and itself (Å.W.-M.). For the purpose of open access, the author has applied a CC-BY public copyright license to the Author Accepted Manuscript version arising from this submission.

## Author contributions

Conceptualization, G.P.S., F.G., and Å.W.-M.; methodology, G.P.S.; formal analysis, G.P.S.; writing – original draft, G.P.S.; writing – review and editing, G.P.S., F.G., and Å.W.-M.; visualization, G.P.S.; supervision, Å.W.-M.; project administration, Å.W.-M.; funding acquisition, G.P.S. and Å.W.-M.

## Declaration of interests

The authors declare no competing interests.

## Supplemental information

Data S1. Arduino UNO code file for 20 Hz 5 ms optogenetic stimulation control, related to step 5
